# Geographic Differences in Element Accumulation in Needles of Aleppo Pines (*Pinus halepensis* Mill.) Grown in Mediterranean Region

**DOI:** 10.3390/molecules24101877

**Published:** 2019-05-15

**Authors:** Michaela Zeiner, Ana Kuhar, Iva Juranović Cindrić

**Affiliations:** 1 Man-Environment-Technology Research Centre, School of Science and Technology, Örebro University, Gymnastikgatan 1, 70182 Örebro, Sweden; 2 Division of Analytical Chemistry, Department of Chemistry, BOKU—University of Natural Resources and Life Sciences, Muthgasse 18, 1190 Vienna, Austria; 3 Department of Chemistry, Faculty of Science, University of Zagreb, Horvatovac 102a, 10000 Zagreb, Croatia; ana.starcevic.89@gmail.com (A.K.); ijuranovic@chem.pmf.hr (I.J.C.)

**Keywords:** alepski bor, Aleppo pine, geographical distribution, ICP-AES, ICP-MS, element content, pine needles

## Abstract

Pine needles are widely used as bio-indicators due to their worldwide distribution and the ease of sample collection. In contrast to deciduous trees, conifers offer the possibility of monitoring long-term exposure through older needles. *Pinus halepensis* Miller is a pine species native to the Mediterranean region, which has been used for restoration activities in arid and semiarid areas leading to vast spatial expansion. Needles from pine trees collected in the southeastern to northwestern extension of Croatia’s coastal area at twelve sampling sites were analysed for twenty-one metals and metalloids. Statistical evaluation of the obtained data revealed significant differences for Al, As, B, Ba, Ca, Cr, Fe, K, Mg, Mn, Na, Se, and Sr between the different regions. Needles from trees growing on islands did not show elevated levels of Mg and/or Na as a result of the sea spray influence. The differences in metal accumulation are supposed to be linked to the environmental conditions at the respective sampling site, since the species was the same everywhere. By comparing the elemental contents of the soil those of with needles, it can be clearly seen, that the root as well as the foliar uptake contribute to the final amount.

## 1. Introduction

The Aleppo pine (*Pinus halepensis* Miller) named after the city Aleppo in Syria is a pine species originating from arid and semiarid areas in the Mediterranean region. Miller describes this pine species as follows: “PINUS (*Halepensis*) foliis geminis tenuissimis, conis obtusis, ramis patulis. [...] Pine-tree with two narrow leaves in each sheath, obtuse cones, and spreading branches. Pinus Halepensis, foliis tenuibus lætè viridibus. […] Aleppo Pine with very narrow dark green leaves.” [1] The tree is small to medium-sized; its slender, light green needles can reach a length up to 12 cm. The reproduction takes place via distribution of seeds by wind and birds. [2] Recent studies have shown that *Pinus halepensis* is a valuable medical plant. Its essential oil has been proven to exhibit nootropic and neuroprotective activities, which supports the treatment of Alzheimer’s disease [3]. Powder of Aleppo needles can be successfully applied to heal skin burns [4]. Extracts of bark, cones and seeds exhibit antibacterial activity [5].

Besides the natural occurrence, this tree has been widely used for reforestation purposes in the 19th and 20th centuries [6,7]. Such plantations are aimed to reduce degradation of (semi)arid areas caused by climatic changes and anthropogenic influences. Especially this pine species, characterised by high-resistance to adverse climatic and soil conditions, is highly estimated as a pioneer species to protect soils against desertification [7]. Consequence of this prevalence of *Pinus halepensis* in afforestation is a vast spatial expansion of this tree, in some parts even with a growth rate of 600% per year [6]. Besides the positive effects of these plantations on soil properties [8], the invasive character of Aleppo pine showed negative effects on floral and faunal biodiversity alongside changes in the hydrological system [6,7]. Due to its distribution along the Croatian coastline, the locally called alepski bor seems to be a good representative plant for studying geographical differences in metal accumulation. Furthermore, Aleppo pines have only been scarcely used in monitoring studies regarding metal accumulation in needles [9,10], data that are more recent are available for tree rings [11,12] or for bark [11,12,13].

Plants or plant parts are widely used for investigating environmental pollution coming from soil, water and/or air. Mosses are found to be more efficient than conifer needles for air quality monitoring [14]. Nevertheless, conifer and especially pine needles present good bio-indicators in environmental monitoring, not only for organic [15], but also for inorganic pollutants [16], whereby various parameters influence the final quantified amount of a metal in the respective specimen. The impact from the growing area must be considered [17], which depends on the local climatic conditions, the soil composition and the respective pollution [18]. Furthermore, characteristics of the tree, for instance its age [19], species and/or hybridisation [20] determine the needle metal contents. Conversely, soil properties—such as acidity—are changed by the pine trees themselves, which subsequently has an impact on the bioavailability and root uptake of certain metals [21].

Apart from the negative impact on plants by metals coming from various pollution sources, certain elements are required for a proper functioning of the plants’ physiology and thus essential nutrients [22].

Thus, the presented investigation covers the following 21 metals and metalloids: aluminium, arsenic, *boron*, barium, *calcium*, cadmium, cobalt, *copper*, chromium, *iron*, *potassium*, lithium, *magnesium*, *manganese*, *molybdenum*, sodium, nickel, lead, selenium, strontium, and *zinc* (plant nutrients according to Plank and Kissel [22] in italic). Regarding the geographical differences to be studied, twelve sampling sites were chosen along the Croatian coastline from northwest down to southeast

## 2. Results

The analytical method used has been proven to be fit for purpose. The figures of merit of both methods, such as recoveries to show the trueness, precision and day-to-day-repeatability to see the influence of random errors are given besides the instrumental conditions in Table 1.

All obtained calibration curves had coefficients of determination (R^2^) higher than 0.999. The determined LOQs are listed in Table 2 alongside the results of the elemental contents in the analysed pine needles. Each sampling site was considered as one population; the respective means were tested by ANOVA for significant differences. Statistically significant dependency of the elemental needle contents on the sampling site (*p* < 0.05) was found for thirteen of the investigated twenty one analytes, namely Al, As, B, Ba, Ca, Cr, Fe, K, Mg, Mn, Na, Se, and Sr. The content ranges of each sampling site as well as the *p*-values from the ANOVA-tests are given in Table 3.

## 3. Discussion

### 3.1. Metal Needle Contents for Pinus halepensis

The chosen plant species *Pinus halepensis* is wide spread along the Croatian coastline, thus it fulfils basic criteria for a plant being used as bio-monitor, namely being represented in large numbers all over the monitoring area besides the easy and economic sampling procedure [23]. The analytical method applied was optimised for the given matrix and the obtained figures of merit were considered acceptable. This conclusion was drawn based on the validation parameters determined, i.e., trueness (expressed as recovery in %), precision (expressed as relative standard deviation - RSD), and day-to-day-repeatability (in %), LOQ (in mg/kg dry matrix) as well as coefficients of determination of the respective calibration curves (data are listed in Table 1 and Table 2 and mentioned in Section 2—results). Since the recoveries obtained were all around 100%, no significant interferences by the matrix is to be expected, meaning that there is no need for matrix-matched standards. The ICP-SFMS employed in the present study is specified for R = 4500 in medium resolution, and R = 11,000 in high resolution. Since spectral separation of ^40^Ar^35^Cl^+^ from ^75^As^+^ requires a minimum resolution of 7770, we have set the instrument to high resolution for measuring As. Furthermore, chloride is always present in digested plant samples leading to an overestimation of As if not properly resolved. Even if sensitivity at high resolution settings is lower than in medium resolution, the limit of detection is not significantly increased due to the lower background and lower background fluctuation.

Furthermore, to ensure trueness of the results, washing of the needles after sampling was performed carefully. Plant material offers the possibility to monitor elements that are naturally occurring as well as resulting from pollution in the environment depending on time and place. Origin of the metals and metalloids found in the needles is on the one hand given by root uptake from soil and on the other hand via uptake through the stomata on the needle surface. Aerial deposition of metals caused by anthropogenic activity is especially high in industrial areas and near roads. In order to exclude the metals only attached to the needle surface from those taken up, needles have to be washed prior to sample preparation to avoid falsification of the results [9].

Even if *Pinus helapensis* is highly abundant in the Mediterranean area and its applicability as a bio-indicator in arid environments has been previously demonstrated [9], few studies have been published dealing with the inorganic needle composition. Data for comparison to the obtained results from the presented study are given in Table 2. The investigation by the Jordanian working group covered only four potentially toxic metals, namely cadmium, copper, lead and zinc [9]. Of all these metals, the contents ranged wider for the Croatian trees than those from Jordan. In this respect, it must be considered that the latter study focused on Amman city and its surroundings, whereas the present paper describes sampling sites along the entire Croatian coastline. With the exception of lead, the contents found are comparable. Higher amounts for lead in the Jordanian needles can be explained by the fact, that leaded petrol was the predominant fuel used by automobiles at the time when the sampling was carried out. The publication by the Catalonian research group includes eight metals, the former four and additionally aluminium, arsenic, chromium and nickel [10]. The results found in this study are in the same order of magnitude, but as stated above the ranges are larger, which can be explained by the wider sampling area. For As, Cd, and Cr the obtained mean content for all the trees in all of the sampling sites is beyond the upper end of the ranges determined for the Spanish Aleppo pine needles. Conversely, for Al the mean is below the lower end of the range for the Catalonian samples [10].

Comparing the elemental contents of the data in needles of *Pinus halepensis* with those of other pine species has to be treated with care, since each species shows different accumulation behaviour, even when grown under the same conditions [24,25]. In general, the results for the major elements, determined by the soil background composition, such as Ca, K, Mg, and Na are always in the g/kg range, whereas the other elements studied are present in the µg/kg to mg/kg range (trace and ultra-trace elements).

### 3.2. Geographical Differences

**Figure 1:**
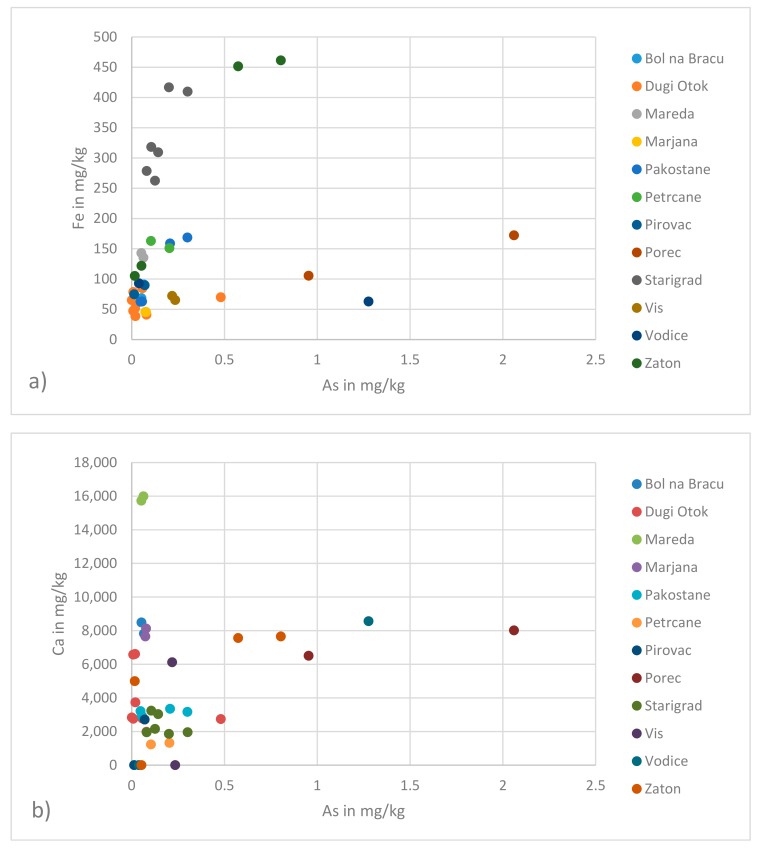
Correlation of needle contents of selected soil background elements: (**a**) Fe vs. As; and (**b**) Ca vs. As.

Arsenic is not only a pollutant given by anthropologenic activity, but also its distribution in soils of agricultural and grazing land is directly related to the underlying geology [26]. Thus, it is not surprising that As is one of the elements whose needle contents differ statistically significantly between the different regions. An overview map on As distribution in European top soils shows the As levels close to sampling site 8 (Poreč) to be the highest. This fact is reflected in the highest As needle contents found for this area (see Table 3). Conversely, a geochemical survey from 2005 showed similar As contents in top and sub soil for the whole country Croatia [27]. In this respect, it must be considered that pine tree roots reach deeper soil horizons and not only the top layer, which has been analysed in the soil survey [26] and furthermore that the dry and wet precipitation also contribute to the metal uptake. Differences in needle and soil contents can easily be seen by plotting As content in needles versus the needle contents of two important soil background elements, namely Ca and Fe (see Figure 1).

Regarding Cd in Croatian top soils, an unequal distribution has been found, with higher values towards the south [28,29]. However, this tendency was less significant in the investigation from 2005 [27]. The resultant accumulation of Cd in the pine needles does not differ statistically significant. Neither for the environmental pollutants Ni and Pb statistically significant difference in the needle contents were found. Furthermore, for these two elements, the soil levels vary throughout the sampled region [27,28]. Similar findings were reached for Co, an element being associated to Ni.

The main rock forming elements, such as Al, Ca, K, Fe, Mg, and Na, were found in the needles in geographically dependent contents. In addition, they all display unequal distribution along the Croatian coastline [28], whereby the variation in the soil content differs from the variation in the needle content. Comparing samples from the island with those from the mainland, no spray influence resulting in higher Na and/or Mg contents could be found, as having been reported for needles from *Pinus canariensis* growing on the Atlantic island Tenerife [30].

The dispersal of flysch zones in the coastal region causes increased strontium soil concentrations, the highest being registered in the interior of Istria [28]. According to this information presented in the geochemical atlas of Croatia by Halamić, highest Sr needles contents are to be expected in the samples from the sampling sites 4 (Marjana), 5 (Pakoštane), 6 (Petrčane), and 7 (Pirovac), which are only given for the Petrčane area. Furthermore, the results for sampling site 3 (Mareda) and 8 (Poreč), which are located on the peninsula Istria, but not in its inner part, are among the highest found in the presented study. Thus, it is assumed that not only the soil composition alone affects the trees’ metal uptake, but also that spreading of certain elements via wind has to be considered.

Barium, especially present in igneous rocks, becomes bioavailable due to weathering processes. In the coastal areas, the soil contents are lower than in the inner part of Croatia. Some islands in the South, such as Brač, Hvar, Korčula and Mlijet, also have elevated Ba content [28]. Despite this element presenting statistically significantly differences between the sampling sites, this trend is not directly reflected in the obtained data.

Chromium, being considered an indicator for basic soil, was found to be present in highest soil level close to Starigrad [28]. The samples with the highest Cr needle contents were collected in this area.

The abundance of the chalcophile trace element Copper is associated with other transitional elements, e.g., As, Co, Cr, Fe, and Ni. Copper in (agricultural) soil is mainly of anthropogenic origin, especially in areas with apple or vine cultivation. Within Croatia, the highest soil Cu levels are found in the coastal region, with an increasing tendency towards south. [28] Maximum Cu content in needles was found for Starigrad (sampling site 9; 13.7 mg/kg), this area having also the highest mean value of all regions studied (6.43 mg/kg), followed by Zaton and Vis (sampling sites 12 and 10; max. 10.3 mg/kg and 8.11 mg/kg, resp.). The high variance within each area led to no statistically significant difference between the sampling spots.

For Manganese, the geochemical atlas of Croatia shows the highest soil contents in central Dalmatia close to sampling sites 7 (Pirovac) and 11 (Vodice), as well as 9 (Starigrad) and 12 (Zaton) [28]. The needle samples from the latter three contained high quantities of Mn, but even less than those from Dugi Otok (sampling site 2) and Bol na Braču (sampling site 1). By comparing Cu with Mn, it can be seen that the variance within the different areas are smaller for the latter, so the values for each area significantly different from each other.

According to Halamić and colleagues [28], increased zinc soil concentrations are characteristic for the Velebit Mountain seaside and the whole central and southern Dalmatia, whereby higher amounts are present in their hinterland than in the coastal area, alongside island Vis. For this place (sampling site) the highest zinc needle contents were found. Otherwise, the results are quite similar resulting in non-statistically significant differences.

The abundance of Li in soil varies. The element has been reported to be present in higher concentrations in arid soils. Lithium in the environment has not been studied to a great extent, due to its low toxicity to plants and animals, but the increasing use and resultant disposal of Li-containing products, e.g., batteries, makes this element worth investigating [31]. The geochemical atlas of Croatia does not cover Lithium. The needle Li content does not differ statistically significantly between the sampling sites. The highest values were found for sampling site 8 (Poreč; >4 mg/kg), followed by Zaton (sampling site 12; max. 2.67 mg/kg), Pakoštane (sampling site 5; max. 2.64 mg/kg), and Petrčane (sampling site 6; max. 2.50 mg/kg).

Boron, being an essential element for plants, is widely distributed in the environment, in both hydrosphere and lithosphere [32]. Its needle contents vary widely, from not detectable values (below LOD) up to 99 mg/kg. Not only does this trend apply to the different areas, but also within the single sampling sites, such as in Zaton (sampling site 12), Dugi Otok (sampling site 2) and Starigrad (sampling site 9) large ranges were found.

Selenium is another element, with paucity of data for Croatian soils. For its importance in sheep fodder, it was studied along with soil analysis in Slavonia and Barania in the eastern part of the country, but not covering the coastal areas [33]. As for boron, statistically significant differences were found, but within each sampling sites, the contents do not vary widely.

Regarding molybdenum, all determined needle contents are considered to belong to one group of data, no statistically significant differences based on the geographical origin of the samples was found. The Mo soil levels are considered relatively high in Croatia due to the long weathering history of karstic residual soils [34].

### 3.3. Pinus halepensis as Medicinal Plant

Aleppo pines are used as medicinal plants for various applications. The most common ones are based on using powder of Aleppo needles, extracts of bark, cones and seeds besides its essential oil. Thus, the present amounts of potentially toxic metals for the plant parts of interest are regulated by the WHO document “Quality control methods for medicinal plant materials” [35]. Limit values are stated for Cd and Pb with 0.3 mg/kg and 10 mg/kg, respectively. Exceedances of these quantities were found for three trees from sampling site 9 (Starigrad), for both Cd and Pb. Furthermore, one tree from sampling site 12 (Zaton) contained more Cd than allowed; 0.85 mg/kg. Pb, besides the above-mentioned exceeding, one tree from Marjana (sampling site 4) and one from Pirovac (sampling site 7), had a high needle content, 25 mg/kg and 13 mg/kg, respectively. Overall, 8.3% of all analysed trees (*n* = 60) are not appropriate for medical use regarding Pb and 6.7% referring to Cd. Thus, their application in general seems to be safe, but it has to be taken into account that for other potentially toxic elements, such as As, Cu, or Ni no maximum contents are stipulated.

## 4. Materials and Methods

### 4.1. Samples

**Figure 2:**
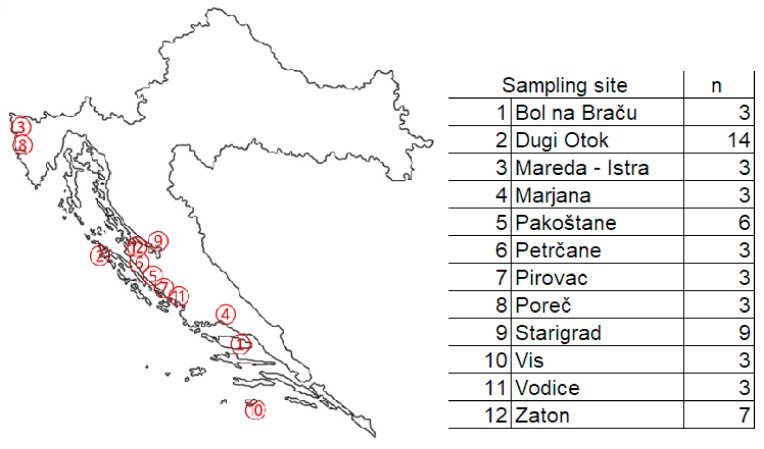
Location of the twelve sampling sites for pine needles in coastal area of Croatia.

The sampling was carried out in autumn 2012. Trees of *Pinus halepensis* from twelve different sampling spots were sampled (see Figure 2), whereby always fully developed 1-year-old pine needles were taken from the external and sunlit part of the crown. The age of the trees ranged from 20 years to 30 years. The number of sampled trees ranged from three to fourteen, the data are given in Figure 2. The collected needles of each tree were pooled for further sample preparation.

### 4.2. Chemicals and Glass/Plastic-ware

Nitric acid (HNO_3_) of p.a. (pro analysis) quality and the ICP Multielement Standard IV (1000 mg/L) were purchased from Merck (Darmstadt, Germany). The latter was used to prepare the calibrations standards in the concentration ranges from 0.05 mg/L to 5.0 mg/L for ICP-AES, and from 0.01 µg/L to 100 μg/L for ICP-MS. Measurements for validation purposes were carried out with the standard reference material SRM 1575a—Trace Elements in Pine Needles—obtained from National Institute of Standards and Technology, Gaithersburg, USA.

All glass- and plastic-ware intended for use were pre-cleaned with semi-concentrated nitric acid. Ultrapure water (>18 MΩ* cm) was produced in-house.

### 4.3. Sample Preparation

All pooled needle samples from each tree were rinsed with ultra-pure water and then brought to constant weight by drying in a laboratory oven at 105 °C. Afterwards, the dried samples were homogenised and ground using a metal-free mortar. Three aliquots with a mass ranging from 0.045 g to 0.075 g, weighed to the nearest 0.1 mg, were taken of each dried pooled needles sample for acid microwave assisted digestion. To each vessel with powdered needles, 5 mL of semi-concentrated HNO_3_ (7 mol/L) were added. The used instrument, a MWS-2 Microwave System Speedwave BERGHOF (Eningen, Germany), ran the following temperature program for digestion: step 1: 150 °C for 10 min, step 2: 160 °C for 10 min and step 3: 190 °C for 20 min. The obtained clear digest solutions were subsequently filled up to a final volume of 10.0 mL with ultrapure water. The SRM as well as the blank solutions underwent the same treatment.

### 4.4. Measurements

Elemental concentrations in all solutions, i.e., digests, blanks and standard solutions, were quantified in triplicate. Simultaneous quantification of the analytes was performed either by inductively coupled plasma atomic emission spectrometry (ICP-AES) or by inductively coupled plasma mass spectrometry (ICP-MS). The choice of method applied was based on the expected concentration of analyte. The summary of the instrumental conditions alongside the analytes is given in Table 1.

### 4.5. Optimisation and Characterisation of the Analytical Method

The digest solutions of the pine needles SRM were measured five times for the replicate data as basis for precision expressed as relative standard deviation (RSD). The trueness of the methods, presented as recovery, was determined using the same solutions and calculated based on the following formula:

(1) $$recovery_{x}\;\mathrm{in}\;\%=\;\frac{{\mathrm{content}}_{x,\;\mathrm{found}}\;\mathrm{in}\;\mathrm{mg}/\mathrm{kg}}{{\mathrm{content}}_{x,\;\mathrm{certified}}\;\mathrm{in}\;\mathrm{mg}/\mathrm{kg}}\cdot 100$$

Furthermore, the day-to-day repeatability was calculated based on analysis of selected samples (*n* = 3) on different days.

The limits of detection (LOD) and the limits of quantitation (LOQ) for all analytes in the given needle matrix were based on eleven blank measurements calculated using 3 σ and 10 σ, respectively.

### 4.6. Calculations and Statistics

All obtained elemental concentrations in the digest solutions were blank corrected prior to calculating the results as contents per dry weight considering dilution steps as well as mass of dried sample. In order to check the hypothesis that there is essentially no difference between the mean content in the needle samples from different sampling sites, ANOVA-tests—based on the 0.05 level of significance—were performed using the data-analysis add-in for Excel 2013. Since not all data obtained were normally distributed, a Kruskal–Wallis-test (non-parametric test) was carried out to prove the outcome of the ANOVA test, using the XLSTAT add-in for Excel.

## 5. Conclusions

The presented study is the first thorough investigation of elemental contents in Aleppo pine needles in Croatia including 21 metals and metalloids to follow environmental pollution. Correlation of the needle contents to soil data revealed that the metal uptake via roots represents only one way for metal to enter plants. An additional important factor is the foliar uptake due to dry and wet precipitation. Various elements from anthropological as well as environmental sources can pass long distances due to translocation processes. Regional differences were found for thirteen of the elements investigated, including on the one hand major elements and trace elements, and on the other hand rock-forming elements and pollutants caused by anthropological activity. Apart from the environmental objective of the study, the safe use of needles in various applications in the form of home remedies was evaluated. Less than 10% of the analysed trees contained Cd and/or Pb in their needles in amounts higher than allowed for medicinal plants according to WHO.

## Figures and Tables

**Table 1:** Instrumental conditions and figures of merit for both analytical methods used.

Parameter	ICP-AES ^1^	ICP-SFMS ^2^
Instrument	Prodigy High Dispersive ICP-AES (Teledyne Leeman, Hudson, NH, USA)	Element 2 ICP-SFMS (Thermo Fisher; Bremen, Germany)
Output power	1100 W	1300 W
Argon flows	Coolant:18 L min^−1^	Coolant: 16 L min^−1^
	Auxiliary: 0.8 L min^−1^	Auxiliary: 0.86 L min^−1^
	Nebuliser: 1 L min^−1^	Nebuliser: 1.06 L min^−1^
Sample flow	1.0 mL min^−1^	100 μL min^−1^
Nebuliser	Pneumatic (glass concentric)	PFA microflow
Spray chamber	Glass cyclonic	PC^3^ cyclonic quartz chamber
Plasma viewing	Axial	-------
Analytes	Al at 396.152 nm ^3^	^75^As^+^ (HR) ^4^ ^98^Mo^+^ (MR) ^7^Li^+^ (LR) ^82^Se^+^ (LR) ^111^Cd^+^ (LR) ^208^Pb^+^ (LR) ^115^In^+^ (internal standard at 1.1 µg/L for all resolution levels)
	B at 208.956 nm	
	Ba at 455.403 nm	
	Ca at 396.847 nm	
	Cd at 214.441 nm	
	Co at 228.615 nm	
	Cr at 267.716 nm	
	Cu at 224.700 nm	
	Fe at 238.204 nm	
	K at 766.491 nm	
	Mg at 280.271 nm	
	Mn at 257.610 nm	
	Mo at 202.030 nm	
	Na at 589.592 nm	
	Ni at 231.604 nm	
	Pb at 220.353 nm	
	Sr at 407.771 nm	
	Zn at 213.856 nm	
Recovery	92%–115%	90%–111%
Precision (RSD)	0.07%–2.3%	0.1%–2.1%
Day-to-day repeatability	<2.9%	<2.7%

^1^ inductively coupled atomic emission spectrometry. ^2^ inductively coupled sector field plasma mass spectrometry. ^3^ wavelengths obtained by line selection for the given matrix. ^4^ HR, high resolution; MR, medium resolution; LR, low resolution with the nominal mass resolutions being 350, 4500 and 10,000.

**Table 2:** Elemental contents in Aleppo pine needles for all sampling sites in mg/kg dry matter alongside literature data.

Element	Method	LOQ	Mean	Min	Max	SD	RSD in %	Amman City Jordan [9]	Catalonia (Spain) [10]
Aluminium	AES	2.9	249	32.7	1,000	184	135		376–1150
Arsenic	MS	0.058	0.244	<LOD	2.06	0.411	59		0.0229–0.180
Boron	AES	1.0	35.4	LOD < x < LOQ	99.2	23.5	150		
Barium	AES	1.1	6.28	<LOD	33.0	6.11	103		
Calcium	AES	0.42	4975	1231	16147	3449	144		
Cadmium	MS	0.0017	0.419	0.020	6.16	1.28	33	0.12–1.50	0.0152–0.186
Cobalt	MS	0.011	1.44	0.018	10.0	3.25	44		
Chromium	MS	0.023	1.26	0.063	10.8	2.21	57		0.113–0.756
Copper	MS	0.022	5.27	1.01	52.7	6.81	77	5.32–16.0	3.363–14.463
Iron	AES	0.19	146	32.9	461	118	124		
Potassium	AES	0.84	3123	548	6052	1663	188		
Lithium	MS	0.027	2.00	0.128	20.7	4.53	44		
Magnesium	AES	0.61	2081	640	3310	690	302		
Manganese	AES	0.87	21.2	6.18	39.5	8.97	236		
Molybdenum	AES	0.13	5.74	0.414	48.8	6.43	89		
Sodium	AES	0.20	1431	187	6291	1622	88		
Nickel	MS	0.019	0.994	0.0574	8.01	1.83	54		0.260–2.588
Lead	MS	0.043	3.03	0.0567	25.0	5.79	52	11.0–75.5	0.469–3.179
Selenium	MS	0.016	0.653	0.0634	1.72	0.404	161		
Strontium	AES	0.17	6.86	1.63	15.5	3.88	177		
Zinc	AES	1.1	27.0	2.23	543	67.8	40	10.0–118	20.204–29.162

**Table 3:** Ranges of elemental contents in Aleppo pine needles for the individual sampling sites in mg/kg dry matter alongside *p*-value of ANOVA-test (elements written in italic means statistically significant difference between the sampling sites).

Element	Bol na Braču	Dugi Otok	Mareda-Istra	Marjana	Pakoštane	Petrčane	Pirovac	Poreč	Starigrad	Vis	Vodice	Zaton	*p*-value ^1^
*Aluminium*	156–264	32.7–1000	193–314	50.4–106	84.4–343	174–705	80.9–82.2	142–240	347–528	112–652	112–156	108–517	1.3 × 10^−16^
*Arsenic*	0.0528–0.0665	<LOD–0.481	0.0521–0.0638	0.0736–0.0778	0.0480–0.300	0.104–0.204	0.0136–0.0703	0.954–2.06	0.0809–0.301	0.218–0.235	0.0394–1.28	0.0167–0.804	0.00059
*Boron*	13.0–14.7	15.7–99.2	<LOD	<LOD	24.5–38.2	35.9–55.1	17.3–21.8	24.9–33.8	0.967–46.7	14.0–39.3	16.6–17.2	13.7–67.3	0.015
*Barium*	5.30–7.40	0.0985–14.4	10.7–13.3	3.00–4.55	1.35–6.48	4.14–7.92	1.34–1.74	5.13–7.20	7.34–33.0	3.16–5.69	4.70–9.68	1.69–6.21	1.9 × 10^−6^
*Calcium*	7824–8528	1980–6612	15733–16147	7477–8127	2915–3710	1231–6264	2710–3154	2263–8013	1858–3238	5011–60122	8560–10567	4980–7656	1.3 × 10^−16^
Cadmium	0.115–0.141	0.0207–0.264	0.131–0.140	0.0197–0.0635	0.0605–0.145	0.134–0.225	0.117–0.165	0.112–0.134	0.162–6.16	0.0383–0.0918	0.0376–0.0992	0.0616–0.854	0.055
Cobalt	<LOD–0.0191	0.0198–5.86	<LOD	<LOD	0.0402–0.159	0.0671–0.134	0.0183–0.0969	<LOD	0.0572–10.0	<LOD	<LOD–0.0395	0.150–0.631	0.46
*Chromium*	0.555–1.29	0.219–0.807	0.553–0.620	0.0635–0.690	0.418–1.43	0.582–1.42	0.532–0.914	0.438–0.935	0.910–10.8	0.174–0.498	0.357–0.615	0.554–2.47	0.015
Copper	2.24–4.29	1.69–7.14	1.68–4.03	3.73–5.56	2.58–6.49	2.62–4.06	3.10–4.29	2.84–4.58	1.94–13.7	1.49–8.11	1.01–2.63	1.50–10.3	0.44
*Iron*	68.4–91.0	32.9–86.7	134–143	45.5–48.8	62.5–169	151–170	74.7–96.8	99.0–172	263–417	65.3–72.1	62.8–92.8	105–461	7.0 × 10^−10^
*Potassium*	2826–2953	647–4457	690–1450	3723–3929	3627–4437	548–4396	4844–5080	783–4785	611–4202	1956–2021	3340–6830	2590–6052	0.00089
Lithium	0.543–0.602	0.128–0.638	0.334–0.564	0.182–0.313	0.241–2.65	1.39–2.50	0.190–0.976	4.20–4.88	0.557–1.02	1.16–1.50	0.125 –0.251	0.159–2.67	0.63
*Magnesium*	2756–2866	1623–3310	1683–2770	2817–3047	1940–2936	1566–1902	1313–1331	1285–1913	1444–1638	2534–3095	3288–4158	640–2756	5.1 × 10^−5^
*Manganese*	31.7–39.5	26.3–36.7	17.7–22.7	6.18–6.33	14.7–19.2	15.6–20.9	10.6–13.8	15.0–15.3	11.2–30.3	12.1–12.8	21.7–23.9	7.93–34.2	3.1 × 10^−8^
Molybdenum	3.68–5.09	0.414–7.35	2.58–4.20	3.80–4.89	3.57–7.36	1.09–4.80	2.66–48.8	4.51–7.58	2.37–16.2	1.87–7.49	2.02–5.42	3.43–10.8	0.093
*Sodium*	924–971	404–1380	1374–1565	187–236	352–1093	4826–5731	711–777	472–870	585–2143	444–598	708–866	1732–6291	1.1 × 10^−11^
Nickel	0.0765–0.301	0.259–2.81	0.363–0.700	0.212–0.987	0.198–0.842	0.113–0.241	0.0778–1.16	0.358–0.411	0.303–8.01	0.0574–0.496	0.113–0.415	0.408–1.35	0.22
Lead	<LOD	0.0969–7.25	<LOD	0.212–25.0	<LOD	0.246–0.443	0.116–13.5	<LOD–0.0954	0.184–11.5	<LOD –0.0574	<LOD	0.0567–1.03	0.65
*Selenium*	0.539–0.682	0.152–0.565	0.317–0.369	1.23–1.24	0.355–1.46	0.649–1.52	0.272–0.878	0.448–0.657	0.503–0.689	0.776–0.955	<LOD–0.0634	0.0800–1.72	0.035
*Strontium*	7.01–7.64	1.63–8.04	9.70–10.2	5.25–5.42	3.71–5.78	10.2–15.5	2.44–2.90	10.3–11.9	10.8–11.5	5.34–6.41	5.53–7.95	3.53–15.2	1.1 × 10^−8^
Zinc	15.2–18.2	2.23–32.6	16.3–18.2	10.9–17.3	16.5–30.4	12.2–60.5	17.3–41.7	11.8–13.4	10.6–29.4	30.3–37.6	11.4–13.5	11.3–18.9	0.99

^1^*p*-values from Kruskal–Wallis-Test: Al 0.004; As < 0.001; B 0.012; Ba < 0.0001; Ca < 0.0001; Cd 0.128; Co 0.112; Cr < 0.0001; Cu 0.394; Fe < 0.0001; K 0.014; Li 0.196; Mg 0.0003; Mn < 0.0001; Mo 0.163; Na < 0.0001; Ni 0.18; Pb 0.539; Se 0.056; Sr < 0.0001; and Zn 0.08.
